# The Effect of Fructooligosaccharide and Inulin Addition on the Functional, Mechanical, and Structural Properties of Cooked Japonica Rice

**DOI:** 10.3390/gels12010048

**Published:** 2026-01-01

**Authors:** Bing Dai, Ruijun Chen, Shiyu Chang, Zheng Wei, Xiaohong Luo, Jiangzhang Wu, Xingjun Li

**Affiliations:** 1 Academy of National Food and Strategic Reserves Administration, National Engineering Research Center for Grain Storage and Transportation, Beijing 102209, China; bingdai3@126.com (B.D.); ruijun_chen@126.com (R.C.); csy@ags.ac.cn (S.C.); wz@ags.ac.cn (Z.W.); lxh@ags.ac.cn (X.L.); 2 College of Grain and Strategic Reserves, Henan University of Technology, Zhengzhou 450001, China; qlsszz@126.com; 3 College of Food Science and Engineering, Wuhan Polytechnic University, Wuhan 430048, China

**Keywords:** inulin, fructooligosaccharide, cooked rice, amylopectin aging, oligofructose, pasting profile

## Abstract

To test whether fructooligosaccharide (FOS) and inulin (INU) molecules can improve the hardness of cooked rice through forming a hydrogel network, we added FOS or INU at 0%, 3%, 5%, 7%, and 10% concentrations to two cooking japonica rice and compared the cooking and textural parameters, the pasting, thermal, and thermo-mechanical properties, and the microstructure of the cooked rice. General Linear Model Univariate (GLMU) analysis revealed that, compared with no oligofructose addition, both FOS and INU addition reduced the rice cooking time and increased the gruel solid loss. The addition of these dietary fibers (DFs) to cooking rice lowered the hardness, adhesiveness, springiness, gumminess, and chewiness of the rice, but maintained the cohesiveness and increased the resilience. Compared with no oligofructose addition, FOS and INU addition improved the smell, taste, and total sensory score of cooked rice. The addition of these DFs significantly decreased the trough, peak, final, breakdown, and setback viscosities, but increased the pasting temperature and peak time. Both FOS and INU addition decreased the enthalpy of gelatinization but increased the peak and conclusion temperature of gelatinization of rice flour paste. After the retrograded flour pastes were kept at 4 °C for 21 days, both FOS and INU significantly increased amylopectin aging compared with no oligofructose addition. The FOS-added and INU-added rice doughs had a higher dough development time and stability time, gelatinization peak torque, setback torque, and gelatinization speed, with a lower protein weakening degree, amylase activity, breakdown torque, heating speed, and enzymatic hydrolysis speed. Compared with no oligofructose addition, both FOS and INU addition reduced the amorphous region of starch and β-sheet percentage, but increased the percentages of random coils, α-helixes, and β-turns in cooked rice. Principal component analysis (PCA) further demonstrated that the gruel solid loss, cooked rice hardness, chewiness, gumminess, taste, and the peak, trough, breakdown, final, and setback viscosities were sensitive parameters for evaluating the effects of species and the amount of oligofructose addition on rice quality. The microstructure showed that FOS or INU addition induced thickening of the matrix walls and an increase in the pore size, forming a soft and evenly swollen structure. These results suggest that FOS or INU addition inhibits amylose recrystallization but maintains amylopectin recrystallization in cooked rice, with INU addition producing greater improvements in the texture and sensory scores of cooked rice compared withFOS addition. This study provides evidence of the advantages of adding DFs and probiotics such as INU and FOS to cooked rice.

## 1. Introduction

Fructooligosaccharides (FOSs) are a blend of 1-kestose (GF2) to fructooligosaccharide (GF7) and fructodisaccharide (F2) to fructooligosaccharide (F8), processed from Jerusalem artichoke or chicory using partial enzyme hydrolysis or membrane separation techniques, purification, and drying, or a blend of 1-kestose (GF2) to kestohexaose (GF5) processed from sucrose by the catalysis of β-fructofuranosidase from *Aspergillus oryzae* or *Aspergillus niger*, followed by separation, purification, and drying [1]. Inulins are linear polysaccharides formed by β-(2→1)-linked fructose units, usually with a terminal glucose residue linked by an α-(1→2) bond (GF_n_-type fructans), or Fn-type fructans lacking the terminal glucose; their degree of polymerization (DP) is usually in the range of 2–60 [2,3]. Commercial inulin polymers are prepared with varying DPs and different amounts of oligosaccharides [4]. Inulins are utilized by probiotics as prebiotics in the human large intestine. They participate in various physiological functions, including promoting beneficial bacterial growth, balancing intestinal flora, regulating glycemia and blood lipid levels, enhancing mineral absorption, and decreasing colon cancer risks [5]. Inulins also have excellent processing behaviors in the food industry and show better water holding capacity, gelation, rheology, and other characteristics compared with ordinary dietary fiber (DF). Thus, inulins are widely added in functional foods and common foods such as dairy products, noodles, beverages, and meat products [6,7]. In recent years, to increase DF intake, many researchers have carried out experiments on the effects of inulins on dough processing performance and the quality of flour products, such as gluten-free dough [4], spaghetti [8], bread [9], plain dough [10], and soft and strong wheat dough [11]. However, few studies have examined the effects of inulin (INU) and FOS addition on cooking rice.

Rice belongs to a very high glycemic-index cereal food [12]. Several studies have displayed that consuming rice grains as a staple food is associated with a high probability of class II diabetes in Southeast Asia and East Asia [13]. Adding DF to food is currently the main approach for supplementing DF intake in the human body. Studies have shown that the hydrolysis rate of starch in wheat flour with added DF is significantly decreased, indicating that this food should be considered a low-glycemic-index food, with health-promoting effects [14]. However, a common method of rice consumption habits is to cook the rice grains and then eat them. Thus, it is worth exploring the effects of the addition of exogenous DF during cooking on the cooked rice product.

China has processed chicory and Jerusalem artichoke root crops since 2000. China’s INU output was less than 1000 tons in 2009, but it sharply increased to 15,000 tons by 2019 and exceeded 22,000 tons in 2023 [15]. China’s inulin market size will reach 0.1 million tons in 2025 and 0.24 million tons in 2030 [15]. The application of INU has extended from traditional dairy and pasta products to healthcare products due to obesity and overweight issues (regulation of intestinal health).

Compared with indica rice, japonica rice is slightly more expensive and, when cooked, it has a better taste and fragrant flavor [16]. Here, we hypothesized that INU and FOS molecules could contribute intermolecular interactions and establish a network structure comparable to a hydrogel, and that adding INU and FOS during the cooking of japonica rice would promote the gelatinization of starch, given that these amorphous molecules have strong hygroscopicity and can produce many hydrogen bonds with rice protein and starch molecules. General Linear Model Univariate (GLMU) analysis in SPSS software (Version 17.0) is suitable for testing the effect of unequivocal independent variables on continuous variables, including interaction effects, main effects, and covariate analysis [17]. To test the above hypotheses, we employed a lower-temperature three-year-stored sea rice variety (SQ) and a newly harvested japonica rice variety (Nanjing 5, NJ5) as the test samples, and used GLMU analysis and Principal Component Analysis (PCA) to compare the cooking characteristics, cooked rice texture profile, flour pasting, and thermal properties of the rice, as well as the thermo-mechanical properties of rice flour doughs and the microstructures of cooked rice kernels. The goal was to improve the eating quality and shelf life of cooked rice and promote the health and sustainable development of the population through the addition of these DFs to rice.

## 2. Results and Discussion

### 2.1. Effect of Adding FOS or Inulin on the Cooking Properties of Japonica Rice

SQ rice had a higher optimal cooking time and water absorption ratio than NJ5 but a lower gruel solid loss (Table 1). Compared with FOS addition, INU increased the rice cooking time and gruel solid loss but decreased the water absorption ratio. Compared with no oligofructose addition, 5–10% addition significantly reduced the rice cooking time, increased the gruel solid loss, and maintained the water absorption ratio. The 5–10% addition of FOS or INU significantly reduced the cooking time of both japonica rice varieties, similar to the reduction in the optimal cooking time of spaghetti observed with the addition of 4% INU with different DPs [18]. These results suggest that the strong hygroscopic nature of inulin might cause the dilution of starch or gluten, thereby accelerating the process of gelatinization.

### 2.2. Effect of Adding FOS or Inulin on the Textural Profiles of Cooked Japonica Rice

Compared with cooked SQ rice, cooked NJ5 rice had a lower hardness, adhesive force, adhesiveness, cohesiveness, springiness, gumminess, and chewiness, possibly because it was a newly harvested rice; however, there was no difference in the resilience of the two varieties (Table 2). Compared with no DF addition, INU addition significantly decreased the hardness, adhesive force, adhesiveness, springiness, gumminess, and chewiness; FOS addition decreased the hardness, gumminess, and chewiness. Compared with FOS addition, cooked rice with INU addition had a lower hardness, adhesive force, adhesiveness, springiness, gumminess, and chewiness, but showed higher resilience. Further, compared with no oligofructose, 3–10% addition significantly decreased the hardness, adhesive force, adhesiveness, springiness, gumminess, and chewiness of cooked rice, but maintained the cohesiveness and resulted in higher resilience. These results are similar to those of a previous study where 4% INU addition with different DPs decreased the sensorial firmness and adhesiveness of cooked spaghetti [18]. However, Barro and Franco [19] reported that 5–10% addition of medium-polymerized INU significantly increased the firmness of bread crumb. Barro and Franco [19] also acknowledged that the water content in bread is directly related to the bread’s softness; the greater the amount of water in fresh bread, the slower the staling [20]. Thus, we argue that INU and FOS bind to water due to being highly soluble, resulting in softer cooked rice and lower chewiness.

### 2.3. Effect of Adding FOS or Inulin on the Sensory Evaluation Parameters of Cooked Japonica Rice

Cooked NJ5 rice had less of an odor and lower palatability, cool rice texture, and total taste score than cooked SQ rice (Table 3). Compared with no DF addition, INU addition improved the odor, palatability, taste, and total score, while maintaining the appearance and cool rice texture. However, FOS decreased the appearance while maintaining or even increasing the odor, taste, cool rice texture, and total score. Compared with FOS addition, INU addition increased the odor, appearance, structure, palatability, and total score of cooked rice. Compared with no oligofructose in cooked rice, 5% addition resulted in the strongest odor and highest cool rice texture and total score. Handa et al. [21] suggested that the addition of 2–25% FOS in baked foods was optimal, with 13.72% FOS resulting in the best acceptability of cookies. The current findings suggest that the addition of 3–10% INU or FOS as a fiber ingredient contributes to an improved mouth feel and improved taste and texture of cooked rice, with INU producing a stronger odor and better appearance, structure, palatability, and total score than FOS.

### 2.4. Effect of Adding FOS or Inulin on the Pasting Parameters of Japonica Rice Flours

Compared with SQ rice flour, the water suspension of NJ5 rice flour exhibited lower peak, final, breakdown, and setback viscosities, and a lower pasting temperature, but had a longer peak time of pasting (Table 4). Compared with no DF addition, both the addition of FOS and INU reduced the peak, trough, breakdown, final, and setback viscosities, and increased the pasting temperature and peak time. Compared with FOS addition, INU addition decreased the peak and final viscosities and increased the pasting temperature, but maintained the trough, breakdown, and setback viscosities and the peak time of pasting. Compared with no oligofructose addition, the addition of 3–10% FOS or INU significantly decreased the peak, trough, breakdown, final, and setback viscosities, and increased the pasting temperature and peak time. These results are similar to the previously observed diminishing effect of adding 2–8% long and short chain INUs into wheat flour on the trough, peak, breakdown, final, and setback viscosities [22]. According to previous research, starch and non-starch polysaccharides compete for water molecules during the pasting process [23], and the formation of hydrogen bonds in starch molecules is inhibited by the interaction between INU and starch molecules, resulting in difficulty forming a double helix structure of straight-chain starch.

The magnitude of setback viscosity, determined by a rapid viscosity analyzer (RVA), is used to show the retrogradation tendency of amylose in a starch paste, whereas for retrograded starch, the endotherm of a differential scanning calorimeter (DSC) offers a quantitative measurement of enthalpy change and transition temperature for the melting of recrystallized amylopectin [24,25]. In the present study, the pasting characteristics of rice flour were improved because the retrogradation of amylose in the starch paste from an amorphous to an ordered structure during cooling was inhibited by the addition of 3–10% FOS or INU.

### 2.5. Effect of Adding FOS or Inulin on the Thermal Parameters of Japonica Rice Flours

The SQ rice flour paste had a higher onset (*T*_o_), peak (*T*_p_), and conclusion (*T*_c_) temperature of gelatinization, and a higher gelatinization peak height than NJ5 rice flour paste, but had a similar enthalpy of gelatinization and lower peak height relative to NJ5 rice flour paste (Table 5). Compared with no DF addition, both FOS and INU addition decreased the gelatinization enthalpy of rice flour paste, with a greater reduction observed with FOS addition. FOS addition increased the onset (*T*_o_), peak (*T*_p_), and conclusion (*T*_c_) temperature of gelatinization, but the addition of INU did not change these parameters.

After the retrograded rice flour pastes were kept at 4 °C for 21 days (Table 6), compared with no DF addition, both FOS and INU addition maintained the enthalpy, onset temperature, and peak height of gelatinization of rice flour paste; FOS addition maintained the peak temperature, increased the conclusion temperature of gelatinization, and decreased the peak width of gelatinization; INU addition reduced the peak temperature of gelatinization and maintained the conclusion temperature and peak width of gelatinization; INU and FOS addition increased the amylopectin aging of rice paste. The addition of 3–7% oligofructose significantly increased the amylopectin aging of rice flour paste. Our recent study showed that 3–10% FOS addition inhibited amylopectin aging in pure rice starch paste, but 3% INU addition significantly increased amylopectin aging and 5–10% INU addition tended to decrease amylopectin aging [25]. These results suggest that FOS has a stronger suppressing ability on amylopectin aging than INU, and high concentrations (≥10%) of FOS and INU can decrease amylopectin aging in rice flour paste.

### 2.6. Effect of Adding FOS or Inulin on the Thermo-Mechanical Parameters of Japonica Rice Dough

A mixolab is a multi-functional grain quality testing instrument that can fully analyze dough properties, protein quality, starch properties, and enzyme activity through the process of simulating dough from mixing to heating and cooling [26]. The SQ rice flour dough had a lower protein weakening degree (C1–Cs), amylase activity (C3/C4), breakdown torque (C3–C4), setback torque (C5–C4), and heating speed (α) than NJ5 rice flour dough, but had a higher peak torque (C3) and gelatinization speed (β) than NJ5 rice flour dough (Table 7). Both doughs had similar dough development time (DDT), stability time (DST), and enzymatic hydrolysis speed (γ).

Compared with no DF addition, FOS and INU addition significantly increased the DDT, stability time, setback torque, and gelatinization speed, but decreased the protein weakening and amylase activity.

Compared with FOS addition, INU addition resulted in a higher DDT, lower protein weakening and peak torque, and reduced heating, gelatinization, and enzymatic hydrolysis speeds. Both rice doughs had similar dough development times, amylase activity, breakdown torque, and setback torque. Compared with no DF addition, the addition of 3–10% INU or FOS increased the DDT, DST, setback torque, and gelatinization speed, but decreased the protein weakening, amylase activity, breakdown torque, heating speed, and enzymatic hydrolysis speed; 3–5% addition increased the peak torque of the rice dough.

Here, the addition of 3–10% FOS or INU increased the DDT and DST of rice flour dough. This is similar to a previous study where 2.5–10% INU addition with a DP of 2–30 and INU with a DP of 10 increased the DDT and DST of strong and weak gluten wheat dough, respectively [11]. Similarly, in another study, the addition of 5–10% INU with a DP ≥ 10 increased the DDT and DST of wheat dough [19]. A possible explanation for these findings is that the strongly hydroscopic INU and FOS molecules bind to water molecules, forming a rigid barrier layer around some starch or protein molecules, preventing them from binding with water molecules. This, in turn, delays the development and stability time of the rice flour dough.

Here, we consider Biliaderis and Prokopowich’s compatibility theory [27] to explain the inhibition of rice starch retrogradation by FOS and inulin. In a water suspension solution of rice flour, small-molecule DFs such as FOS and INU are compatible with starch. These small-molecule DFs will form a hydrated layer and reduce the starch molecular chain rearrangement, thus inhibiting the starch setback viscosity. However, in rice dough, the structures between small-molecule DFs, such as FOS and INU, and starch are incompatible. These oligofructoses can accelerate the starch recrystallization process; thus, the starch setback torque is not inhibited with increasing addition amounts.

### 2.7. Effect of Adding FOS or Inulin on the Starch Crystallinity and Protein Conformation of Cooked Japonica Rice

Cooked SQ rice had fewer starch amorphous regions (R_1022/995_), more starch crystallinity regions (R_1047/1022_), and a stronger interaction between starch and protein (R_1068/1022_) than cooked NJ5 rice (Table 8). Compared with no oligofructose addition, FOS addition did not change the R_1022/995_ value but decreased the R_1047/1022_ value; INU addition decreased the R_1022/995_ value but did not change the R_1047/1022_ and R_1068/1022_ values, suggesting that FOS decreased the regions of starch crystallinity, but INU decreased the amorphous regions of starch. Both oligofructoses did not change the interaction between starch and protein.

Cooked SQ rice had a higher percentage of random coils and a lower β-sheet percentage than cooked NJ5 rice (Table 8). Compared with no oligofructose addition, FOS addition increased the percentages of α-helixes, random coils, and β-turns in cooked rice, but decreased the β-sheet percentage; INU addition increased the percentages of random coils and α-helixes in cooked rice, but decreased the β-sheet percentage; both INU and FOS addition increased the percentages of α-helixes, random coils, and β-turns in cooked rice, but decreased the β-sheet percentage. β-sheets are formed by peptide chains connected by hydrogen bonds to form sheet-like structures. A decrease in β-sheets indicates that the overall rigidity of the protein has decreased [29]. α-helixes are formed by the amino acid chain through hydrogen bonds to form a helical structure. An increase in α-helixes indicates a decrease in protein stability and an increase in the proportion of random coils and β-turns in proteins [30]. The decrease in the overall rigidity and stability of glutelin in cooked rice might be related to an increase in the gelatinization speed and a decrease in the hardness of cooked rice.

### 2.8. Effect of Rice Variety, DF Species, and Addition Amount on Cooked Rice Using Principal Component Analysis (PCA)

The present study used the PCA method [17] to screen the sensitive measured parameters for evaluating cooked rice with added DF. The first step is that a component plot in rotated space gives the differences among group factors such as rice varieties, DF species, or addition amounts. The second step is that a scree plot shows which measured parameters are grouped together and with are scattered. The scattered parameters are considered very sensitive.

**Figure 1:**
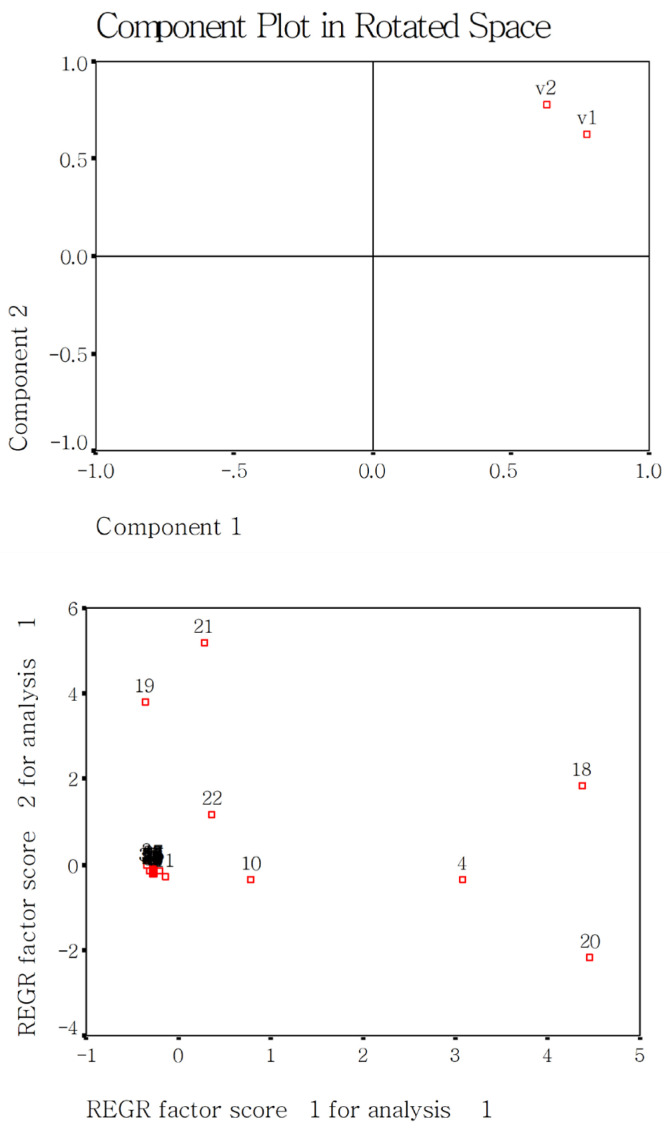
Effect of rice varieties on the measured fifty-four parameters in the present study using PCA analysis. Notes: v1, SQ rice; v2, NJ5 rice; 1, Cooking time; 2, Water absorption rate; 3, Gruel solid loss; 4, Cooked rice hardness; 5, Adhesive force; 6, Adhesiveness; 7, Resilience; 8, Cohesiveness; 9, Springiness; 10, Gumminess; 11, Chewiness; 12, Cooked rice smell; 13, Appearance structure; 14, Palatability; 15, Taste; 16, Cool rice texture; 17, Total taste score; 18, Peak viscosity; 19, Trough viscosity; 20, Break viscosity; 21, Final viscosity; 22, Setback viscosity; 23, Peak time; 24, Pasting temp; 25, Enthalpy of gelatinization—0 d; 26, *T*_o_—0 d; 27, *T*_p_—0 d; 28, *T*_c_—0 d; 29, Peak width—0 d; 30, Peak height—0 d; 31, Enthalpy—21 d; 32, *T*_o_—21 d; 33, *T*_p_—21 d; 34, *T*_c_—21 d; 35, Peak width—21 d; 36, Peak height—21 d; 37, Aging—21 d; 38, DDT; 39, DST; 40, C_3_; 41, C1—Cs; 42, C3—C4; 43, C3/C4; 44, C5—C4; 45, α; 46, β; 47,γ; 48, R_1022/995_; 49, R_1047/1022_; 50, R_1068/1022_; 51, β-sheets; 52, Random coils; 53, α-helixes; 54, β-turns.

Figure 1 presents a loading plot of the principal components after varimax rotation. The two rice varieties were separated, suggesting a significant effect of the rice variety on the physicochemical and quality parameters of rice. Further, a factional scatter-plot of the principal components of the measured fifty-four parameters was constructed. Cooking time (1), cooked rice hardness (4), gumminess (10), peak viscosity (18), trough viscosity (19), break viscosity (20), final viscosity (21), and setback viscosity (22) showed dispersed distributions, but the other 46 parameters were grouped together. These results indicate that the above eight parameters are very sensitive for evaluating the cooked quality of the two japonica rice varieties.

**Figure 2:**
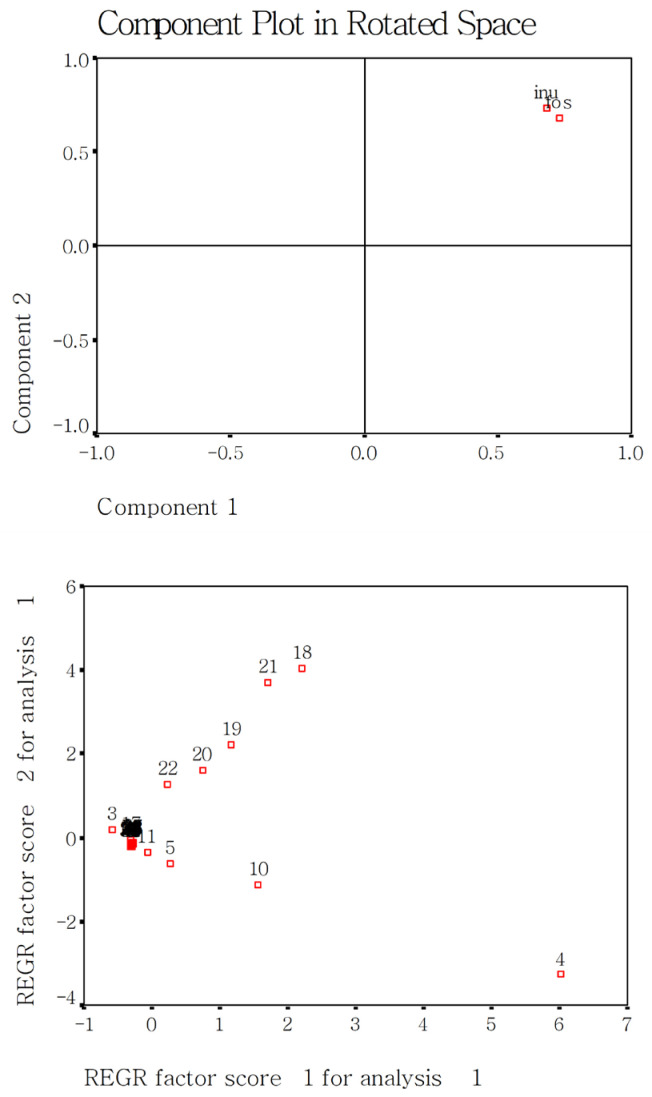
Effect of DF species on the measured fifty-four parameters in the present study using PCA analysis. Notes: fos, FOS; inu, INU; 1–54 are the same as those in Figure 1.

Figure 2 shows the effect of DF species on the measured 54 parameters of the two cooked japonica rice varieties. The effects of DF species (FOS and INU) on rice were not hugely different. The 10 sensitive parameters were gruel solid loss (3), cooked rice hardness (4), adhesive force (5), gumminess (10), chewiness (11), peak viscosity (18), trough viscosity, break viscosity (20), final viscosity (21), and setback viscosity (22).

**Figure 3:**
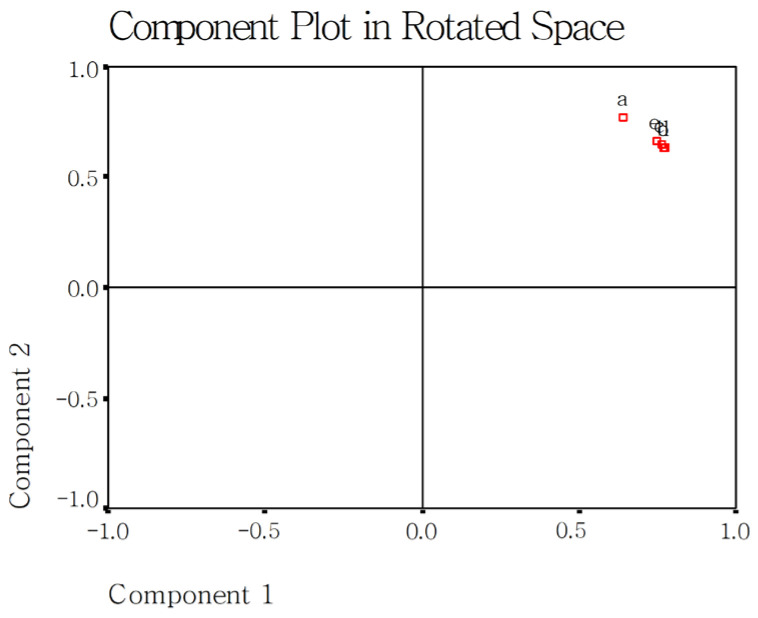

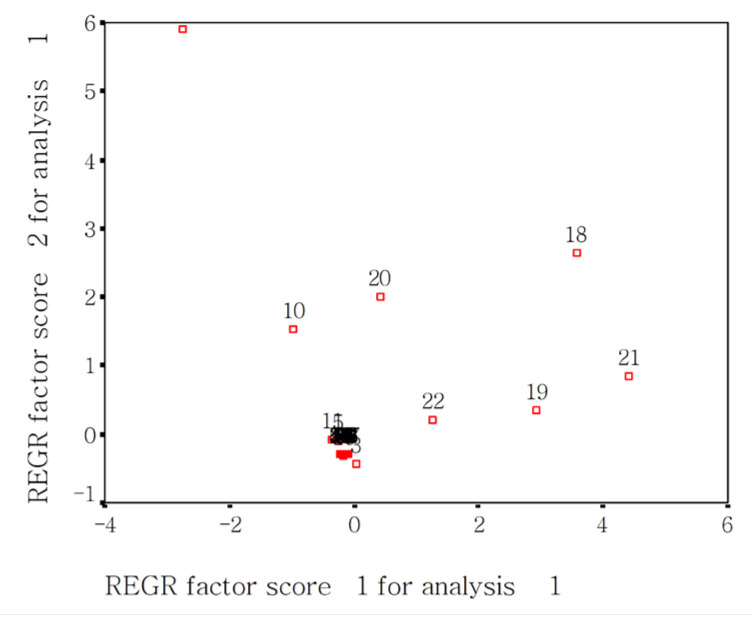
Effect of DF addition on the measured fifty-four parameters in the present study using PCA analysis. Notes: a, 0% DF addition; b, 3% DF addition; c, 5% DF addition; d, 7% DF addition; e, 10% DF addition; 1–54 are the same as those in Figure 1.

Figure 3 shows the effect of DF addition amount on the 54 measured parameters of the two cooked japonica rice varieties. The effect of DF addition amount (0, 3, 5, 7, and 10%) on rice did not show large variation. The eight sensitive parameters were gruel solid loss (3), gumminess (10), taste (15), peak viscosity (18), trough viscosity (19), break viscosity (20), final viscosity (21), and setback viscosity (22).

### 2.9. Effect of Adding FOS or Inulin on the Microstructure of Cooked Japonica Rice

#### 2.9.1. The Surface Microstructure

**Figure 4:**
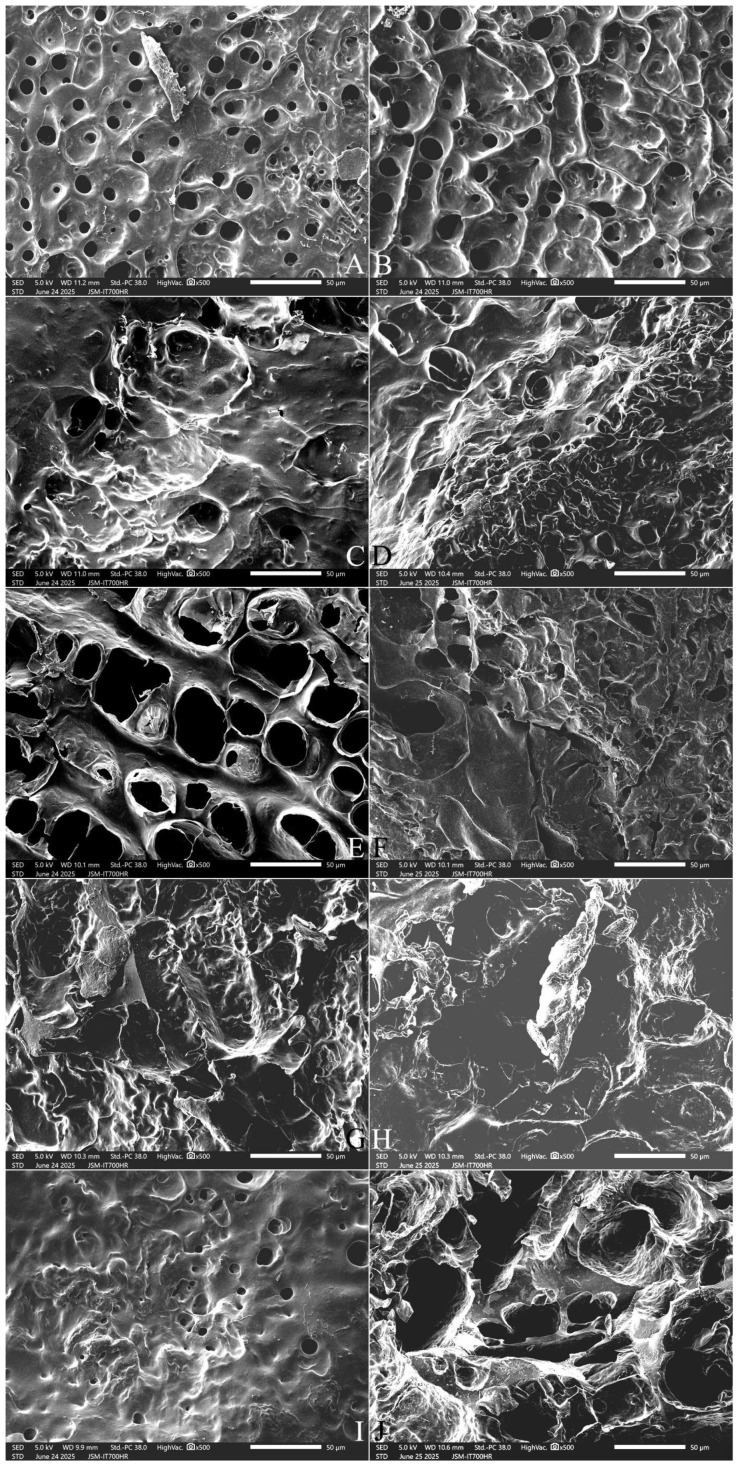
Effect of adding FOS or inulin on the surface microstructure of cooked SQ rice. Notes: (**A**,**B**) are the control samples; (**C**,**E**,**G**,**I**) are the 3%, 5%, 7%, and 10% FOS addition samples, respectively; (**D**,**F**,**H**,**J**) are the 3%, 5%, 7%, and 10% INU addition samples, respectively. All photos are enlarged 500×.

Many small, round holes were left on the surface of the cooked SQ rice. These were formed due to the escape of water molecules during freeze-drying of the samples (Figure 4A,B). Compared with the control sample, 3% FOS addition resulted in larger, sparser holes in the kernel surface (Figure 4C), and 5% FOS addition produced even larger, denser holes (Figure 4E). The addition of 7–10% FOS resulted in pore stacking and an uneven surface (Figure 4G,I). The addition of 3–7% INU induced large, sparse holes in the kernel surface (Figure 4D,F,H), and 10% INU addition resulted in large areas of deep holes (Figure 4J).

**Figure 5:**
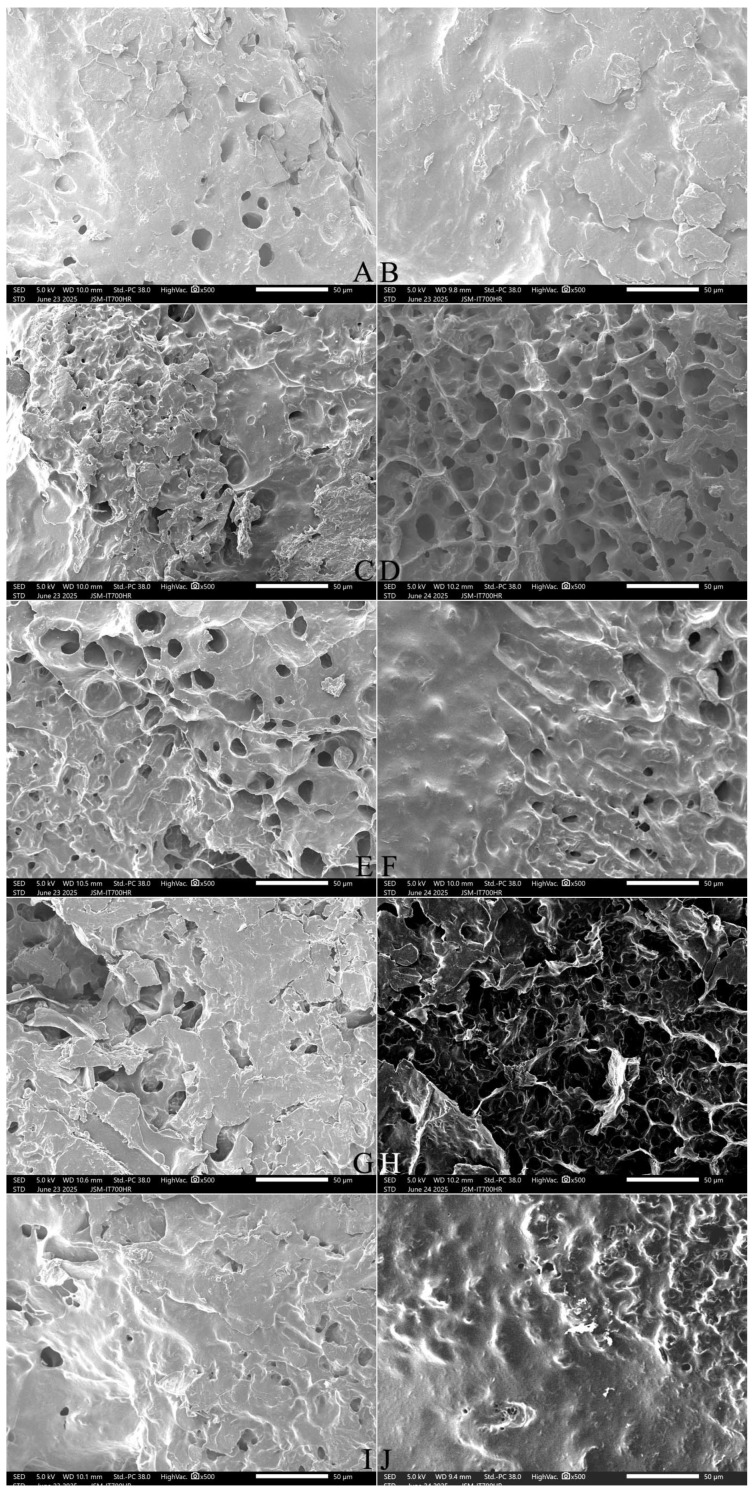
Effect of adding FOS or inulin on the surface microstructure of cooked NJ5 rice. Notes: (**A**,**B**) are the control samples; (**C**,**E**,**G**,**I**) are the 3%, 5%, 7%, and 10% FOS addition samples, respectively; (**D**,**F**,**H**,**J**) are the 3%, 5%, 7%, and 10% INU addition samples, respectively. All photos are enlarged 500×.

Compared with cooked NJ5 rice with no FOS or INU addition (Figure 5A,B), 3%, 5%, and 7% FOS or INU addition resulted in more round holes in the kernel surface (Figure 5C–H). The addition of 10% FOS or INU produced a rough and less integrated surface structure (Figure 5I,J). These results are similar to those of a previous study of the surface of cooked pasta, where 5% INU resulted in an amorphous substance covering the pasta, and 20% INU led to the appearance of many holes in the pasta surface structure, indicating a less integrated structure [31]. These findings could be attributed to greater protein-fiber interactions, allowing FOS and INU to disrupt the compact protein–starch matrix in cooked rice.

#### 2.9.2. The Cross-Section Microstructure

**Figure 6:**
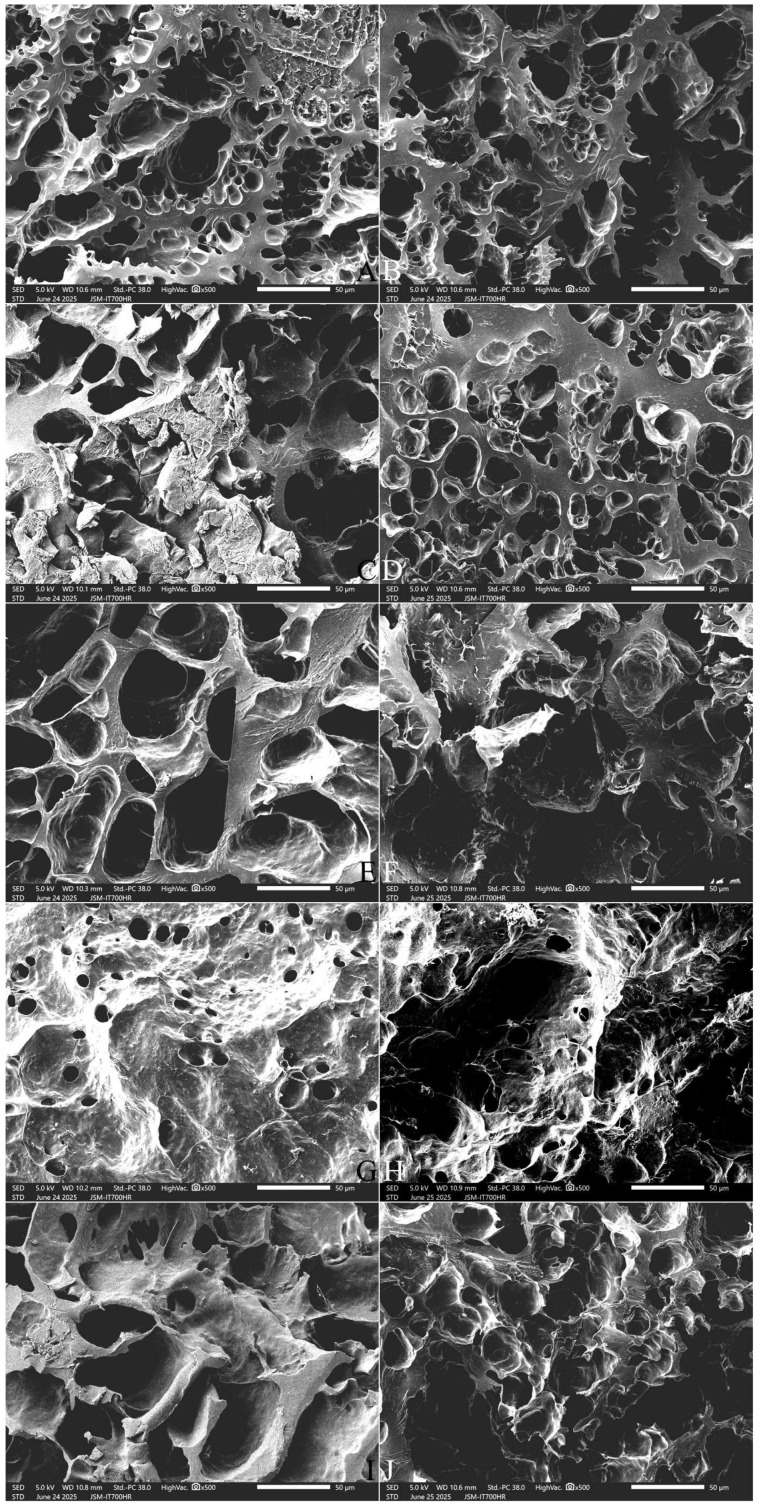
Effect of adding FOS or inulin on the cross-section microstructure of cooked SQ rice. Notes: (**A**,**B**) are the control samples; (**C**,**E**,**G**,**I**) are the 3%, 5%, 7%, and 10% FOS addition samples, respectively; (**D**,**F**,**H**,**J**) are the 3%, 5%, 7%, and 10% INU addition samples, respectively. All photos are enlarged 500×.

**Figure 7:**
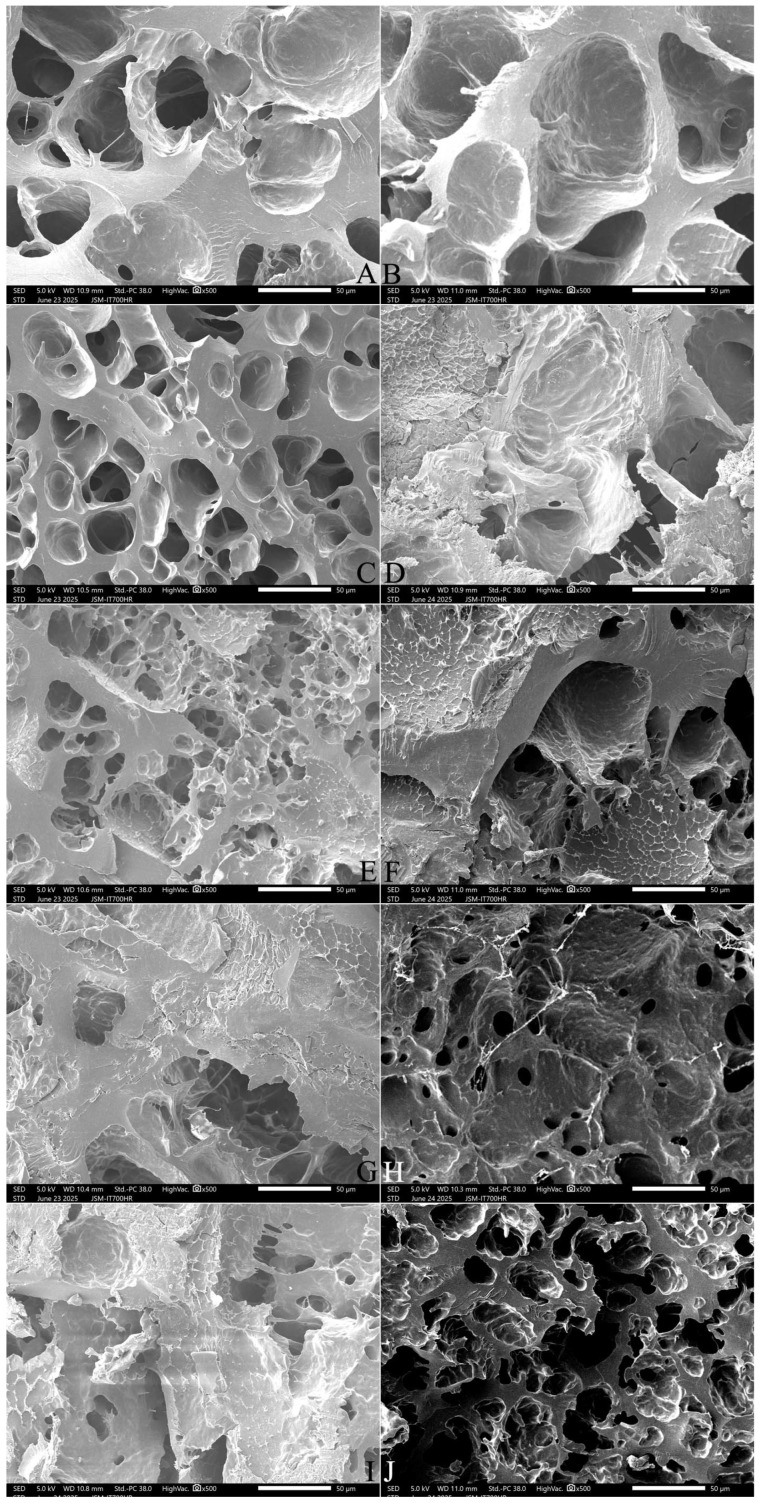
Effect of adding FOS or inulin on the cross-section microstructure of cooked NJ5 rice**.** Notes: (**A**,**B**) are the control samples; (**C**,**E**,**G**,**I**) are the 3%, 5%, 7%, and 10% FOS addition samples, respectively; (**D**,**F**,**H**,**J**) are the 3%, 5%, 7%, and 10% INU addition samples, respectively. All photos are enlarged 500×.

Cooked rice kernels exhibited cracks in the cross-section because of the entry of water molecules. The relatively stale SQ variety (Figure 6A,B) exhibited shallower and larger cracks and much more filament aggregates than the fresh NJ5 variety (Figure 7A,B). The fresh NJ5 rice displayed narrower and deeper cracks that extended into the deep endosperm tissue (Figure 7A,B). The addition of FOS and INU appeared to cause thickening of the matrix walls and an increase in the pore size (Figure 6C–F and Figure 7C–F). With increasing FOS and INU addition, this thickening became less pronounced (Figure 6G–J and Figure 7G–J). These results suggest that FOS and INU promoted the admittance of water molecules into the rice kernel, which enhanced the swelling of starch granules, loosened their internal structure, and produced an open structure in the central area of the cross-section. FOS and INU addition caused a thickening of the matrix walls, possibly by increasing amylopectin recrystallization.

#### 2.9.3. The Longitudinal Section Microstructure

**Figure 8:**
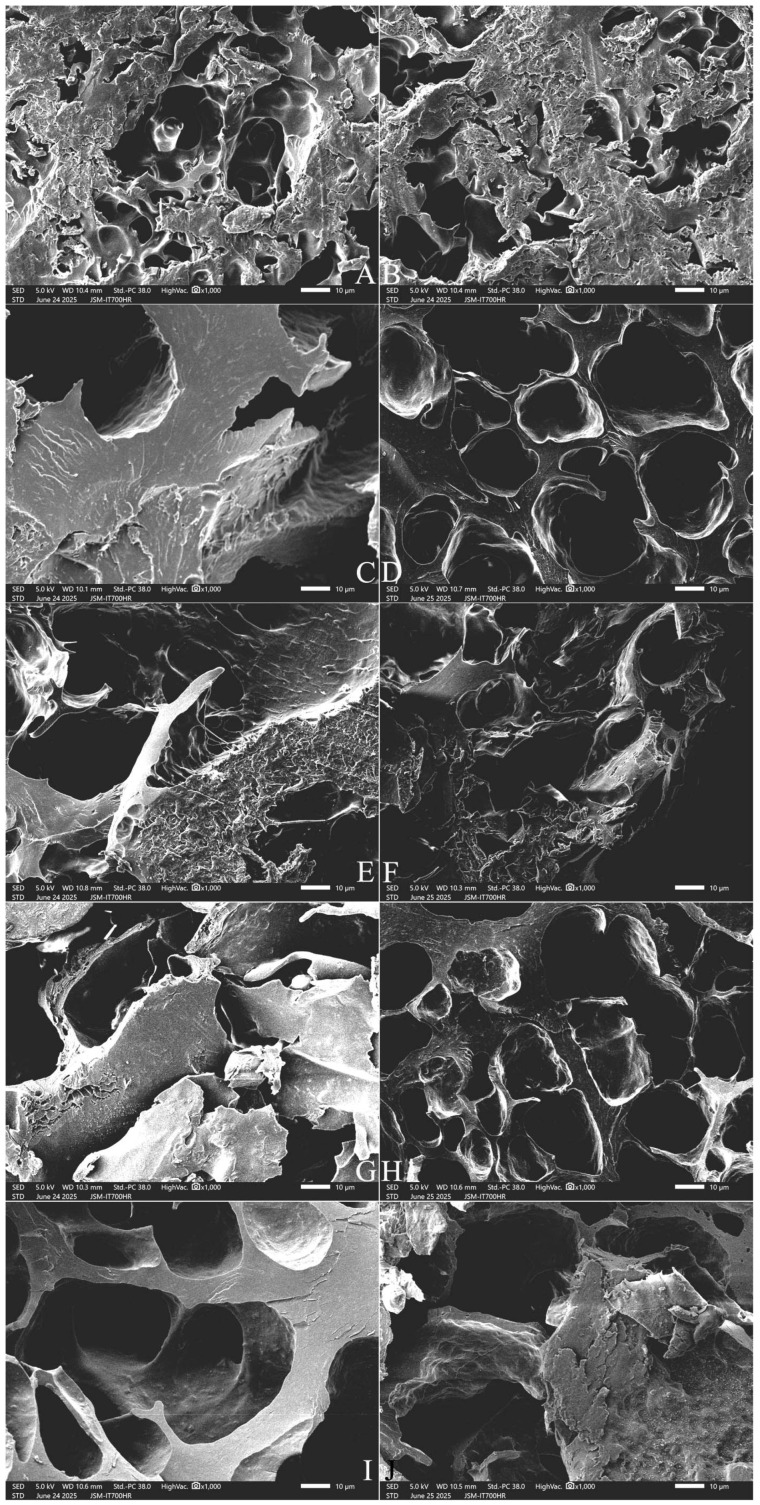
Effect of adding FOS or inulin on the longitudinal-section microstructure of cooked SQ rice**.** Notes: (**A**,**B**) are the control samples; (**C**,**E**,**G**,**I**) are the 3%, 5%, 7%, and 10% FOS addition samples, respectively; (**D**,**F**,**H**,**J**) are the 3%, 5%, 7%, and 10% INU addition samples, respectively. All photos are enlarged 1000×.

**Figure 9:**
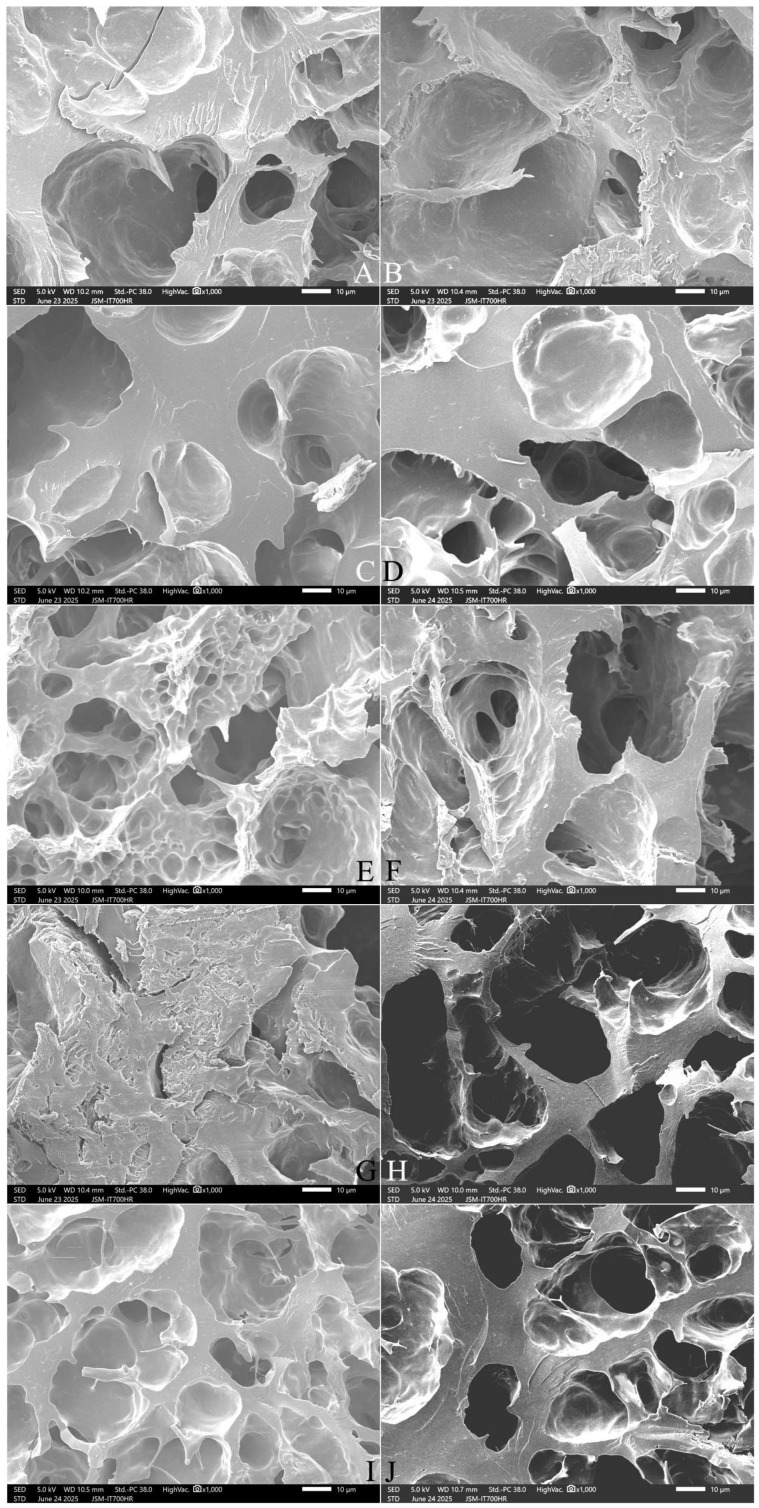
Effect of adding FOS or inulin on the longitudinal-section microstructure of cooked NJ5 rice. Notes: (**A**,**B**) are the control samples; (**C**,**E**,**G**,**I**) are the 3%, 5%, 7%, and 10% FOS addition samples, respectively; (**D**,**F**,**H**,**J**) are the 3%, 5%, 7%, and 10% INU addition samples, respectively. All photos are enlarged 1000×.

Figure 8 and Figure 9 show that SQ rice had more pores and filaments in the longitudinal section (Figure 8A,B), whereas NJ5 rice had larger pores and thicker starch bodies in the cooked grains (Figure 9A,B). With an increase in the amount of added FOS, from 3% to 7%, thickening of the matrix walls and an increase in the pore size was observed (Figure 8C,E,G and Figure 9C,E,G). With the addition of 10% FOS, NJ5 rice (Figure 9I) formed deeper, softer, fresher honeycomb structures than SQ rice (Figure 8I).

With an increase in the amount of added INU from 3% to 7%, the honeycomb structure was slowly destroyed, resulting in larger, thicker pores and a reduction in the degree of crystallization; the rice grains were transformed into an evenly swollen and soft structure (Figure 8D,F,H and Figure 9D,F,H). With the addition of 10% INU, both rice varieties formed deeper, thickened-wall honeycomb structures (Figure 8J and Figure 9J).

Similar to the effects of adding polydextrose to the same varieties of milled rice [32], both FOS and INU addition decreased the chewiness and hardness of cooked rice, reduced the peak and breakdown viscosities of flour water suspension with increases in the pasting temperature and peak time, decreased the enthalpy of gelatinization of flour paste with increases in the peak temperature of gelatinization, and decreased the protein weakness degree of dough with increases in the DDT and DST. Polydextrose, FOS, and INU all decreased the setback viscosities of the flour water suspensions, suggesting that they inhibit amylose recrystallization. Polydextrose addition decreased amylopectin aging in flour paste and setback torque in rice dough [32], but FOS and INU addition increased these parameters. These differences might be due to the different hygroscopicity of these three soluble DFs. At physiological conditions of 37 °C and 0.98 water activity, the equilibrium moisture contents of adsorption and desorption predicted by our measured polynomial equation were 69.43% and 64.09% wet basis for polydextrose [33], respectively, but 35.14% and 37.09%, and 35.71% and 37.7%, for FOS and INU, respectively [25]. In a gelatinized paste of rice flour or a gelatinized dough of rice flour, polydextrose formed a thick hydrated layer and reduced the rearrangement of long-chain amylopectins, but FOS and INU did not form a thick hydrated layer and increased amylopectin recrystallization. The Fourier transform infrared spectroscopy (FTIR) analysis further showed that polydextrose, FOS, and INU addition all reduced the amorphous regions of starch (R_1022/995_) in cooked rice, but polydextrose and INU did not change the crystallinity regions of starch (R_1047/1022_), whereas FOS reduced them.

β-sheets exhibit higher mechanical strength and stability in the stretching direction due to their lamellar structure and inter-strand hydrogen bonding; α-helixes, linked by intra-spiral hydrogen bonding and disulfide bonds, provide elasticity and recoverability [29,30,34]. Consistent with polydextrose addition, FOS and INU addition increased the β-sheet percentage but decreased the percentages of α-helixes, random coils, and β-turns in cooked rice. This might have contributed to the lower hardness and chewiness of the cooked rice.

This study is the first to use principal components analysis (PCA) to demonstrate that gumminess and the peak, trough, breakdown, final, and setback viscosities are common sensitive parameters for evaluating the effects of rice varieties, oligofructose species, and addition amount on rice quality. The cooking time and cooked rice hardness also distinguished the two rice varieties, SQ and NJ5. The gruel solid loss, cooked rice hardness, adhesive force, and chewiness were also sensitive indicators for distinguishing FOS and INU addition; gruel solid loss and taste were found to be good indicators of the added amounts of the two DFs. Thus, we conclude that the cooking properties, pasting profile, and textural parameters are very important for evaluating rice quality. Our future work will determine the molecular weight and species of sugars in the gruel solid loss. The hygroscopicity and the FTIR characteristic peaks of the lyophilized samples of FOS-added or INU-added cooked rice will be determined.

## 3. Conclusions

The present study is the first to compare the effect of FOS and INU addition on the cooking properties, textural profile, pasting, thermal, and thermo-mechanical properties, and the microstructure of two japonica rice varieties using GLMU analysis. Both FOS and INU addition reduced the cooking time, hardness, gumminess, and chewiness of cooked rice, the peak, trough, breakdown, final, and setback viscosities of rice flour water suspension, the enthalpy of gelatinization, torque of gelatinization peak, and starch breakdown in rice dough, and the β-sheet percentage of cooked rice. FOS induced the greatest reductions in the cooking time and enthalpy of gelatinization, whereas INU induced the greatest reductions in the hardness, chewiness, and gumminess of cooked rice, the peak and final viscosities, the gelatinization peak torque, and the β-sheet percentage. Both FOS and INU addition induced the same reductions in the trough, breakdown, and setback viscosities, and the starch breakdown torque.

Both FOS and INU addition increased the gruel solid loss, resilience, taste, pasting temperature, and peak time, as well as the peak temperature of gelatinization, amylopectin aging, DDT, DST, protein weakness degree, setback torque, gelatinization speed, and the percentages of α-helixes, random coils, and β-turns. FOS addition induced the greatest increases in the peak temperature of gelatinization, protein weakness degree, gelatinization speed, and percentages of random coils and β-turns. INU addition induced the greatest increases in the gruel solid loss, resilience, pasting temperature, and DDT. Both FOS and INU addition induced the same increases in taste, pasting peak time, amylopectin aging, DST, setback torque, and α-helix percentage. PCA further demonstrated that gumminess, and the peak, trough, break, final, and setback viscosities are common sensitive parameters for evaluating the effect of rice variety, species, and the amount of oligofructose addition on rice quality. These results are useful for producing functional cooked rice with oligofructose addition. Further work will determine the effect of FOS and INU addition on the pasting profile and thermal properties of glutinous rice.

## 4. Materials and Methods

### 4.1. Rice Samples

The newly acquired japonica rough rice sample of the “Nanjing 5” (NJ5) variety was obtained from Zhangjiagang Grain Reserve, Jiangsu Province, China. The “Super Qianhao” (SQ) variety was a japonica rough rice preserved in a 2.41-ton silo at ≤20 °C temperature over three summers. The silo was located in the pilot platform of the Academy of National Food and Strategic Reserves Administration (ANFSRA), Beijing, China, and the bulk temperature was controlled during oversummering using circulating water in the silo walls. The two rice samples had the moisture contents (MCs) of 12.01% and 12.30%, respectively (Table 9). The FOS and INU samples, with respective moisture contents of 2.49% and 4.25%, were supplied by Shanghai Runloy Biotechnology Co., Ltd., Shanghai, China. The rough rice samples were milled for 30 s in a LT 5588 rice polisher (Grain Instrument Co., Ltd., Taizhou City, China) into white rice in the laboratory. The white rice was pulverized into flour with a high-speed pulverizer (Kewei Yongxing Instrument Factory, Beijing, China). FOS and INU at weight ratios of 0%, 3%, 5%, 7% and 10% were added to the rice grains or flour to get the samples. The rice grains were used for cooking tests, whereas the rice flours were used for pasting, thermal, and thermo-mechanical analyses.

In Table 9, the moisture content (MC) in the samples was obtained according to the AOAC method [35]. The kernel length/width ratio was measured using appearance quality scanning equipment for rice [32]. The taste value was determined using a rice taste meter, as described by Liu et al. [32]. The amylose content in rice was gained using the GB/T 15683-2008 method [36]. The protein content in rice was detected according to ISO 14891-2002 [37]. The fatty acid value in rice flour was determined according to the GB/T 5510-2024 standard [38].

### 4.2. Cooking Time, Gruel Solid Loss, and Water Uptake Ratio

The rice cooking time was measured according to Liu et al. [32]. Two grams of milled rice was soaked for 5 min in 20 mL of deionized water in a glass test tube (20-cm length and 2.5-cm diameter) and then cooked in a boiling water bath on an electromagnetic cooker. The minimal cooking time was determined by extracting single rice kernels at 31 s intervals during cooking and pressing each kernel on a glass plate using the back of a spoon head until no white core was observed in any grain. Based on the minimal cooking time, the gruel solid loss and water uptake ratio in cooked rice samples were obtained using the method of Liu et al. [32]. In a boiling water bath, the rice samples (each 2 g) in three parallel measurements were cooked in 20 mL of deionized water in a test tube for a minimal cooking time. The resulting gruel was moved into glass Petri dishes (2.3-cm height and 15.5-cm diameter). The cooked rice was repeatedly washed using deionized water to extract the soluble solids attached to the kernel surface. The combined extracts were dried at 111 °C for 20 h in an electrothermostatic oven. The dried solids were weighed with a balance (0.0001 g), and the gruel solids loss was calculated. Gruel solid loss reflects the leaking of amylose molecules from starch granules into the cooking water during the rice cooking process [39].

### 4.3. Textural Parameters of Cooked Rice

The cooked rice was detected using a CTX textural device (Brookfield, Middleboro, MA, USA). The rice was cooked according to GB/T 15682-2008 [40]. FOS or INU was added to the rice grain samples at mass ratios of 0% to 10%, with the total weight of rice grains plus the DF being 15 g. Distilled water (19.5 g) was then added. The ratio of the sample to distilled water was 1:1.30. The samples were firstly soaked for 30 min at room temperature and subsequently steamed for 40 min in a boiling water pot. The samples were subsequently braised for 20 min and then analyzed using the cylindrical probe (P35) of the texture analyzer. The parameters were set as follows: the pre-test, test, and post-test speedswere2 mm/s; the compression distance was 15 mm with a force of 5 g.

### 4.4. Pasting Parameters

The RVA–TecMaster device (Perten-Ruihua Instrument Co., Ltd., Beijing, China) was utilized to measure the mixed samples of rice flour and DF, according to the GB/T24852-2010 standard [41]. During measurement, the stirring paddle speed was firstly set at 960 r/min for the first 10 s, then reduced to 160 r/min within 20 s, and was finally set at 150 r/min. Initially, the temperature was set at 50 °C for 1.0 min, then increased to 95 °C over 3.69 min and held there for 2.5 min, before being lowered to 50 °C over 2.81 min and maintained for 2 min. The viscosity curve of the pasting was analyzed using RVA–special TCW software (Version 3.17.5.515).

### 4.5. Thermal Parameters

Rice flour and FOS or INU were uniformly mixed, and aliquots of the sample were utilized for gelatinization parameter analysis in a DSC 214 differential scanning calorimeter (Netzsch-GmbH, Freistaat Bayern, Germany) according to the method of Liu et al. [32]. After gelatinization, the samples were kept in small bags at 4 °C for 21 days and then analyzed for retrogradation. The aging of the retrograded flour paste was calculated using Equation (1).

Aging(%) = (Enthalpy of gelatinization measured at day 21)/(Enthalpy of gelatinization measured at day 0) × 100(1)

### 4.6. Thermo-Mechanical Parameters

A Mixolab device (Chopin-technologies, Tripette et Renaud, Paris, France) was used to analyze the thermo-mechanical parameters of rice dough, as described by Wang et al. [26]. All assays were performed at 60% water hydration.

### 4.7. Fourier Transform Infrared Spectroscopy (FTIR)

The FTIR spectra in the cooked rice specimens were obtained using a Nicolet 6700 FTIR device (Thermo-Fisher Scientific, Waltham, MA, USA), according to the method described by Liu et al. [32]. The samples were pestled with KBr and then made into tablets. The scanning wavenumber was set at 400–4000 cm^−1^. The wavenumber ratios R_1068/1022_ indicate the ratio between protein and starch; R_1047/1022_ and R_1022/995_ indicate the short-range of the starch granule surface, showing the crystalline and amorphous regions of starch, respectively [27]. The secondary structures of the proteins were determined mainly from the spectral variations in the amide I zone. The important protein structures in the amide I region include: β-turns (1660 to 1700 cm^−1^), α-helixes (1650 to 1660 cm^−1^), random coils (1640 to 1650 cm^−1^), and β-sheets (1600 to 1640 cm^−1^).

### 4.8. Sensory Assessment of Cooked Rice

The cooked rice was assessed using the GB/T 15682-2008 method [40] and the procedure of Liu et al. [32]. The first-order indicator score (100) includes smell (20), appearance structure (20), palatability (30), flavor (25), and cool cooked-rice texture (5). Eight trained evaluators (4 males and 4 females) aged between 23 and 26 years observed, tasted, ate, and evaluated the cooked rice using the hedonic scaling test. The evaluator was instructed not to smoke and eat food one hour before the tasting test, but could drink water. During the tasting session, the evaluator maintained a natural physiological state without utilizing cosmetics or other products with intense odors. The tasting test was conducted either one hour before a meal or two hours after a meal.

### 4.9. Scanning Electron Microscopy (SEM)

The cooked rice samples were cooled down to room temperature (RT) and stored in a −19.5 °C refrigerator. The samples were freeze-dried in a freeze dryer before SEM observation. The dried samples were prepared and observed using a scanning electron microscope, according to the procedure described by Wang et al. [26]. Each sample was photographed at 100 to 3000 times magnification under an accelerating voltage of 25-kV.

### 4.10. Data Analysis

The data were analyzed using SPSS (Version 18.0, SPSS Incorporated, Chicago, IL, USA [42]). The samples of raw milled rice were compared using *t*-tests. To examine the main effects of rice variety, DF species, and addition level, General Linear Model Univariate (GLMU) analysis was performed, and the LSD test was used to compare means. Statistical significance was set at *p* < 0.05. Data reduction and factor method was used for principal component analysis and to create the scatter-plots.

## Figures and Tables

**Table 1:** Effect of adding FOS or inulin on the cooking properties of japonica rice.

Factor	Levels	Cooking Time (min)	Water Absorption Ratio	Gruel Solid Loss (mg/g)
Rice	SQ	19.939 ± 0.054 ^a^	3.003 ± 0.047 ^b^	73.256 ± 2.007 ^d^
variety	NJ5	17.989 ± 0.054 ^g^	2.871 ± 0.047 ^c^	87.224 ± 2.007 ^c^
DF	FOS	18.839 ± 0.054 ^de^	3.163 ± 0.047 ^a^	67.838 ± 2.007 ^e^
species	INU	18.988 ± 0.054 ^c^	2.711 ± 0.047 ^d^	92.642 ± 2.007 ^b^
Addition	0	19.357 ± 0.085 ^b^	2.928 ± 0.075 ^bc^	47.025 ± 3.173 ^f^
(%)	3	19.230 ± 0.085 ^b^	2.797 ± 0.075 ^cd^	66.234 ± 3.173 ^e^
	5	18.928 ± 0.085 ^cd^	3.042 ± 0.075 ^ab^	77.956 ± 3.173 ^d^
	7	18.733 ± 0.085 ^ef^	3.019 ± 0.075 ^b^	91.833 ± 3.173 ^bc^
	10	18.571 ± 0.085 ^f^	2.897 ± 0.075 ^bc^	118.153 ± 3.173 ^a^

Notes: SQ, Super Qianhao; NJ5, Nanjing 5; DF, dietary fiber; FOS, fructooligosaccharide; INU, Inulin. All data are expressed as mean ± SD; number of trial repetitions: *n* = 3 for water absorption ratio and gruel solid loss and *n* = 4 for cooing time; each rice variety, each DF species, and each DF addition amount separately had 30, 30, and 12 data points for the GLMU analysis of the water absorption ratio and gruel solid loss, and 45, 45, and 16 data points for the GLMU analysis of cooking time. Different superscript letters indicate significant differences (*p* < 0.05) within the same column for each parameter.

**Table 2:** Effect of adding FOS or inulin on the textural parameters of cooked japonica rice.

Factor	Levels	Hardness (g)	Adhesive force(g)	Adhesiveness (mJ)	Resilience (0.1)	Cohesiveness (0.1)	Springiness (mm)	Gumminess (g)	Chewiness (mJ)
Rice	SQ	2354 ± 76 ^b^	106 ± 5 ^b^	2.60 ± 0.19 ^b^	1.33 ± 0.04 ^abc^	3.03 ± 0.04 ^a^	9.21 ± 0.24 ^a^	707 ± 23 ^b^	67.8 ± 3.3 ^b^
variety	NJ5	1477 ± 76 ^e^	84 ± 5 ^cd^	1.78 ± 0.19 ^c^	1.27 ± 0.04 ^bcd^	2.91 ± 0.04 ^bc^	7.47 ± 0.24 ^d^	431 ± 23 ^f^	32.8 ± 3.3 ^e^
DF	FOS	2155 ± 76 ^c^	119 ± 5 ^a^	2.83 ± 0.19 ^ab^	1.25 ± 0.04 ^cd^	2.96 ± 0.04 ^abc^	9.10 ± 0.24 ^a^	640 ± 23 ^c^	59.8 ± 3.3 ^c^
species	INU	1676 ± 76 ^d^	72 ± 5 ^e^	1.55 ± 0.19 ^c^	1.34 ± 0.04 ^ab^	2.98 ± 0.04 ^ab^	7.58 ± 0.24 ^cd^	498 ± 23 ^de^	40.8 ± 3.3 ^d^
Addition	0	3011 ± 121 ^a^	128 ± 8 ^a^	3.11 ± 0.30 ^a^	1.17 ± 0.06 ^d^	2.97 ± 0.06 ^abc^	9.60 ± 0.38 ^a^	892 ± 36 ^a^	89.7 ± 5.2 ^a^
(%)	3	1564 ± 121 ^de^	77 ± 8 ^de^	1.41 ± 0.30 ^c^	1.42 ± 0.06 ^a^	3.00 ± 0.06 ^ab^	7.88 ± 0.38 ^bcd^	474 ± 36 ^def^	39.1 ± 5.2 ^de^
	5	1748 ± 121 ^d^	103 ± 8 ^b^	2.57 ± 0.30 ^ab^	1.27 ± 0.06 ^bcd^	3.02 ± 0.06 ^a^	8.20 ± 0.38 ^bc^	529 ± 36 ^d^	43.8 ± 5.2 ^d^
	7	1567 ± 121 ^de^	75 ± 8 ^de^	1.46 ± 0.30 ^c^	1.33 ± 0.06 ^abc^	2.87 ± 0.06 ^c^	7.56 ± 0.38 ^cd^	446 ± 36 ^ef^	35.9 ± 5.2 ^de^
	10	1687 ± 121 ^d^	94 ± 8 ^bc^	2.41 ± 0.30 ^b^	1.31 ± 0.06 ^abc^	3.00 ± 0.06 ^ab^	8.46 ± 0.38 ^b^	504 ± 36 ^de^	42.9 ± 5.2 ^d^

Notes: SQ, Super Qianhao; NJ5, Nanjing 5; DF, dietary fiber; FOS, fructooligosaccharide; INU, Inulin. All data are expressed as mean ± SD; number of trial repetitions: *n* = 3, and each rice variety, each DF species, and each DF addition amount separately had 30, 30, and 12 data points for the GLMU analysis. Different superscript letters indicate significant differences (*p* < 0.05) within the same column for each parameter.

**Table 3:** Effect of adding FOS or inulin on the taste score of cooked japonica rice.

Factor	Levels	Smell (%)	Appearance Structure (%)	Palatability (%)	Taste (%)	Cool Rice Texture (%)	Total Score (%)
Rice	SQ	17.54 ± 0.21 ^a^	17.36 ± 0.15 ^ab^	23.96 ± 0.27 ^ab^	22.06 ± 0.18 ^b^	4.34 ± 0.06 ^ab^	85.26 ± 0.55 ^ab^
variety	NJ5	16.86 ± 0.21 ^c^	17.26 ± 0.15 ^bc^	23.32 ± 0.27 ^c^	22.02 ± 0.18 ^b^	4.16 ± 0.06 ^c^	83.62 ± 0.55 ^cd^
DF	FOS	16.82 ± 0.21 ^c^	16.98 ± 0.15 ^c^	23.06 ± 0.27 ^c^	21.98 ± 0.18 ^b^	4.26 ± 0.06 ^bc^	83.10 ± 0.55 ^d^
species	INU	17.58 ± 0.21 ^a^	17.64 ± 0.15 ^a^	24.22 ± 0.27 ^a^	22.10 ± 0.18 ^b^	4.24 ± 0.06 ^bc^	85.78 ± 0.55 ^a^
Addition	0	16.80 ± 0.34 ^c^	17.50 ± 0.24 ^ab^	23.40 ± 0.43 ^bc^	21.50 ± 0.29 ^c^	4.10 ± 0.10 ^c^	83.30 ± 0.86 ^cd^
(%)	3	17.25 ± 0.34 ^abc^	17.35 ± 0.24 ^abc^	24.05 ± 0.43 ^ab^	21.50 ± 0.29 ^c^	4.15 ± 0.10 ^c^	84.30 ± 0.86 ^bcd^
	5	17.60 ± 0.34 ^a^	17.30 ± 0.24 ^abc^	24.20 ± 0.43 ^ab^	22.00 ± 0.29 ^bc^	4.50 ± 0.10 ^a^	85.60 ± 0.86 ^ab^
	7	16.85 ± 0.34 ^bc^	17.45 ± 0.24 ^ab^	23.55 ± 0.43 ^abc^	22.45 ± 0.29 ^ab^	4.25 ± 0.10 ^bc^	84.55 ± 0.86 ^abc^
	10	17.50 ± 0.34 ^ab^	16.95 ± 0.24 ^c^	23.00 ± 0.43 ^c^	22.75 ± 0.29 ^a^	4.25 ± 0.10 ^bc^	84.45 ± 0.86 ^abcd^

Notes: SQ, Super Qianhao; NJ5, Nanjing 5; DF, dietary fiber; FOS, fructooligosaccharide; INU, Inulin. All data are expressed as mean ± SD; number of trial repetitions: *n* = 8, and each rice variety, each DF species, and each DF addition amount separately had 80, 80, and 32 data points for the GLMU analysis. Different superscript letters indicate significant differences (*p* < 0.05) within the same column for each parameter.

**Table 4:** Effect of adding FOS or inulin on the pasting parameters of japonica rice flours.

Factor	Levels	Peak Viscosity (cp)	Trough Viscosity (cp)	Breakdown Viscosity (cp)	Final Viscosity (cp)	Setback Viscosity (cp)	Peak Time (min)	Pasting Temp (°C)
Rice	SQ	4586 ± 13 ^a^	2298 ± 28 ^bc^	2289 ± 35 ^a^	3581 ± 25 ^b^	1284 ± 5 ^a^	5.81 ± 0.02 ^f^	74.46 ± 0.08 ^a^
variety	NJ5	3339 ± 13 ^g^	2262 ± 28 ^bc^	1077 ± 35 ^h^	3356 ± 25 ^e^	1095 ± 5 ^f^	6.33 ± 0.02 ^a^	71.69 ± 0.08 ^g^
DF	FOS	4009 ± 13 ^d^	2308 ± 28 ^b^	1702 ± 35 ^d^	3501 ± 25 ^c^	1194 ± 5 ^c^	6.08 ± 0.02 ^cd^	72.99 ± 0.08 ^e^
species	INU	3916 ± 13 ^e^	2252 ± 28 ^bc^	1664 ± 35 ^de^	3436 ± 25 ^d^	1184 ± 5 ^c^	6.07 ± 0.02 ^cd^	73.17 ± 0.08 ^d^
Addition	0	4549 ± 20 ^b^	2399 ± 44 ^a^	2149 ± 55 ^b^	3683 ± 40 ^a^	1284 ± 7 ^a^	5.93 ± 0.03 ^e^	72.32 ± 0.13 ^f^
(%)	3	4162 ± 20 ^c^	2296 ± 44 ^bc^	1866 ± 55 ^c^	3549 ± 40 ^bc^	1254 ± 7 ^b^	6.02 ± 0.03 ^d^	72.53 ± 0.13 ^f^
	5	3994 ± 20 ^d^	2401 ± 44 ^a^	1593 ± 55 ^ef^	3571 ± 40 ^b^	1169 ± 7 ^d^	6.14 ± 0.03 ^b^	73.10 ± 0.13 ^d^
	7	3737 ± 20 ^f^	2228 ± 44 ^c^	1509 ± 55 ^f^	3364 ± 40 ^e^	1136 ± 7 ^e^	6.11 ± 0.03 ^bc^	73.45 ± 0.13 ^c^
	10	3371 ± 20 ^g^	2074 ± 44 ^d^	1296 ± 55 ^g^	3177 ± 40 ^f^	1102 ± 7 ^f^	6.16 ± 0.03 ^b^	73.99 ± 0.13 ^b^

Notes: SQ, Super Qianhao; NJ5, Nanjing 5; DF, dietary fiber; FOS, fructooligosaccharide; INU, Inulin. All data are expressed as mean ± SD; number of trial repetitions: *n* = 3, and each rice variety, each DF species, and each DF addition amount separately had 30, 30, and 12 data points for the GLMU analysis. Different superscript letters indicate significant differences (*p* < 0.05) within the same column for each parameter.

**Table 5:** Effect of adding FOS or inulin on the thermal parameters of cooked japonica rice at day 0.

Factor	Levels	Δ*H* (J/g)	*T*_o_ (°C)	*T*_p_ (°C)	*T*_c_ (°C)	Peak Width (°C)	Peak Height (0.01 mw/mg)
Rice	SQ	6.435 ± 0.072 ^cd^	63.837 ± 0.358 ^a^	70.750 ± 0.317 ^a^	77.493 ± 0.215 ^a^	6.687 ± 0.136 ^d^	12.990 ± 0.314 ^a^
variety	NJ5	6.472 ± 0.072 ^c^	59.415 ± 0.358 ^e^	67.383 ± 0.317 ^e^	74.720 ± 0.215 ^d^	7.333 ± 0.136 ^a^	11.601 ± 0.314 ^d^
DF	FOS	6.272 ± 0.072 ^e^	62.373 ± 0.358 ^b^	69.550 ± 0.317 ^b^	76.293 ± 0.215 ^b^	6.843 ± 0.136 ^cd^	12.462 ± 0.314 ^abc^
species	INU	6.636 ± 0.072 ^b^	60.878 ± 0.358 ^cd^	68.583 ± 0.317 ^cd^	75.920 ± 0.215 ^bc^	7.177 ± 0.136 ^ab^	12.127 ± 0.496 ^bcd^
Addition	0	7.158 ± 0.115 ^a^	60.400 ± 0.567 ^d^	68.017 ± 0.501 ^de^	75.533 ± 0.340 ^c^	7.233 ± 0.214 ^ab^	12.932 ± 0.496 ^ab^
(%)	3	6.661 ± 0.115 ^b^	62.333 ± 0.567 ^b^	69.533 ± 0.501 ^b^	76.458 ± 0.340 ^b^	7.108 ± 0.214 ^abc^	13.174 ± 0.496 ^a^
	5	6.265 ± 0.115 ^de^	62.058 ± 0.567 ^b^	69.508 ± 0.501 ^b^	76.350 ± 0.340 ^b^	6.775 ± 0.214 ^cd^	12.041 ± 0.496 ^bcd^
	7	6.263 ± 0.115 ^de^	61.567 ± 0.567 ^bc^	69.008 ± 0.501 ^bcd^	76.050 ± 0.340 ^bc^	7.033 ± 0.214 ^abcd^	11.933 ± 0.496 ^cd^
	10	5.920 ± 0.115 ^f^	61.777 ± 0.567 ^bc^	69.267 ± 0.501 ^bc^	76.142 ± 0.340 ^bc^	6.901 ± 0.214 ^bcd^	11.394 ± 0.496 ^d^

Notes: SQ, Super Qianhao; NJ5, Nanjing 5; DF, dietary fiber; FOS, fructooligosaccharide; INU, Inulin. Δ*H*, gelatinization enthalpy; *T*_o_, *T*_p_, and *T*_c_ were the onset temperature, peak temperature, and conclusion temperature of gelatinization, respectively. All data are expressed as mean ± SD; number of repetitions: *n* = 3, and each rice variety, each DF species, and each DF addition amount separately had 30, 30, and 12 data points for the GLMU analysis. Different superscript letters indicate significant differences (*p* < 0.05) within the same column for each parameter.

**Table 6:** Effect of adding FOS or inulin on the thermal parameters of cooked japonica rice at day 21.

Factor	Levels	Δ*H* (J/g)	*T*_o_ (°C)	*T*_p_ (°C)	*T*_c_ (°C)	Peak Width (°C)	Peak Height (0.01 mw/mg)	Aging (%)
Rice	SQ	1.780 ± 0.072 ^d^	47.793 ± 1.185 ^a^	58.477 ± 0.210 ^a^	63.833 ± 0.635 ^a^	9.423 ± 0.129 ^ab^	2.728 ± 0.102 ^d^	21.414 ± 0.915 ^c^
variety	NJ5	2.275 ± 0.072 ^b^	48.917 ± 1.185 ^a^	55.490 ± 0.210 ^f^	60.847 ± 0.635 ^c^	9.291 ± 0.129 ^ab^	3.621 ± 0.102 ^b^	31.003 ± 0.915 ^a^
DF	FOS	1.982 ± 0.072 ^c^	49.227 ± 1.185 ^a^	57.153 ± 0.210 ^cd^	62.991 ± 0.635 ^ab^	9.190 ± 0.129 ^b^	3.161 ± 0.102 ^c^	26.131 ± 0.915 ^b^
species	INU	2.075 ± 0.072 ^c^	47.483 ± 1.185 ^a^	56.813 ± 0.210 ^d^	61.690 ± 0.635 ^c^	9.529 ± 0.129 ^a^	3.188 ± 0.102 ^c^	26.286 ± 0.915 ^b^
Addition	0	1.913 ± 0.113 ^cd^	46.733 ± 1.873 ^a^	57.467 ± 0.332 ^bc^	61.350 ± 1.003 ^c^	9.550 ± 0.204 ^a^	2.945 ± 0.162 ^cd^	23.312 ± 1.447 ^c^
(%)	3	2.678 ± 0.113 ^a^	49.892 ± 1.873 ^a^	56.192 ± 0.332 ^e^	64.033 ± 1.003 ^a^	9.542 ± 0.204 ^a^	4.125 ± 0.162 ^a^	32.783 ± 1.447 ^a^
	5	2.121 ± 0.113 ^bc^	49.567 ± 1.873 ^a^	56.850 ± 0.332 ^cde^	63.808 ± 1.003 ^a^	9.608 ± 0.204 ^a^	3.215 ± 0.162 ^c^	26.932 ± 1.447 ^b^
	7	2.032 ± 0.113 ^c^	46.892 ± 1.873 ^a^	56.708 ± 0.332 ^de^	61.617 ± 1.003 ^bc^	9.442 ± 0.204 ^ab^	3.194 ± 0.162 ^c^	26.940 ± 1.447 ^b^
	10	1.396 ± 0.113 ^e^	48.692 ± 1.873 ^a^	57.701 ± 0.332 ^b^	60.892 ± 1.003 ^c^	8.642 ± 0.204 ^c^	2.393 ± 0.162 ^e^	21.075 ± 1.447 ^c^

Note: SQ, Super Qianhao; NJ5, Nanjing 5; DF, dietary fiber; FOS, fructooligosaccharide; INU, Inulin. Δ*H*, gelatinization enthalpy; *T*_o_, *T*_p_, and *T*_c_ were the onset temperature, peak temperature, and conclusion temperature of gelatinization, respectively. All data are expressed as mean ± SD; number of repetitions: *n* = 3, and each rice variety, each DF species, and each DF addition amount separately had 30, 30, and 12 data points for the GLMU analysis. Different superscript letters indicate significant differences (*p* < 0.05) within the same column for each parameter.

**Table 7:** Effect of adding FOS or inulin on the thermo-mechanical properties of japonica rice dough.

Factor	Levels	DDT(min)	DST(min)	C1–Cs (0.1 Nm)	C3(Nm)	C3/C4
Rice	SQ	1.349 ± 0.054 ^de^	1.700 ± 0.212 ^b^	5.45 ± 0.35 ^c^	1.868 ± 0.006 ^a^	1.220 ± 0.015 ^e^
variety	NJ5	1.365 ± 0.054 ^cd^	1.783 ± 0.212 ^b^	6.54 ± 0.35 ^b^	1.756 ± 0.006 ^d^	1.278 ± 0.015 ^ab^
DF	FOS	1.254 ± 0.054 ^ef^	1.760 ± 0.212 ^b^	6.49 ± 0.35 ^b^	1.825 ± 0.006 ^b^	1.264 ± 0.015 ^bc^
species	INU	1.460 ± 0.054 ^bc^	1.723 ± 0.212 ^b^	5.50 ± 0.35 ^c^	1.799 ± 0.006 ^c^	1.234 ± 0.015 ^cde^
Addition	0	1.112 ± 0.086 ^g^	0.867 ± 0.335 ^c^	10.20 ± 0.57 ^a^	1.795 ± 0.010 ^c^	1.307 ± 0.023 ^a^
(%)	3	1.193 ± 0.086 ^fg^	1.567 ± 0.335 ^b^	6.07 ± 0.57 ^bc^	1.832 ± 0.010 ^b^	1.282 ± 0.023 ^ab^
	5	1.348 ± 0.086 ^cdef^	1.308 ± 0.335 ^bc^	6.22 ± 0.57 ^bc^	1.827 ± 0.010 ^b^	1.267 ± 0.023 ^abcd^
	7	1.527 ± 0.086 ^ab^	2.542 ± 0.335 ^a^	4.07 ± 0.57 ^d^	1.817 ± 0.010 ^b^	1.221 ± 0.023 ^de^
	10	1.605 ± 0.086 ^a^	2.425 ± 0.335 ^a^	3.40 ± 0.57 ^d^	1.789 ± 0.010 ^c^	1.170 ± 0.023 ^f^
Factor	Levels	C3–C4(0.1 Nm)	C5–C4(0.1 Nm)	α(−0.01 Nm)	β(0.01 Nm)	γ(−0.01 Nm)
Rice	SQ	3.26 ± 0.20 ^d^	7.72 ± 0.19 ^c^	8.03 ± 0.15 ^de^	30.99 ± 0.52 ^a^	5.49 ± 0.48 ^abc^
variety	NJ5	3.78 ± 0.20 ^a^	8.11 ± 0.19 ^ab^	9.59 ± 0.15 ^a^	28.77 ± 0.52 ^c^	5.46 ± 0.48 ^abc^
DF	FOS	3.72 ± 0.20 ^abc^	7.91 ± 0.19 ^bc^	9.18 ± 0.15 ^b^	30.66 ± 0.52 ^a^	6.21 ± 0.48 ^a^
species	INU	3.32 ± 0.20 ^cd^	7.91 ± 0.19 ^bc^	8.45 ± 0.15 ^c^	29.10 ± 0.52 ^bc^	4.74 ± 0.48 ^c^
Addition	0	4.04 ± 0.31 ^a^	7.12 ± 0.31 ^d^	9.43 ± 0.23 ^ab^	26.80 ± 0.82 ^d^	6.47 ± 0.76 ^a^
(%)	3	3.95 ± 0.31 ^a^	7.73 ± 0.31 ^bcd^	9.13 ± 0.23 ^b^	30.02 ± 0.82 ^abc^	4.30 ± 0.76 ^c^
	5	3.84 ± 0.31 ^ab^	8.49 ± 0.31 ^a^	9.38 ± 0.23 ^ab^	30.37 ± 0.82 ^ab^	5.82 ± 0.76 ^abc^
	7	3.23 ± 0.31 ^bcd^	7.89 ± 0.31 ^abc^	8.30 ± 0.23 ^cd^	30.93 ± 0.82 ^a^	6.10 ± 0.76 ^ab^
	10	2.55 ± 0.31 ^e^	8.29 ± 0.31 ^ab^	7.82 ± 0.23 ^e^	31.28 ± 0.82 ^a^	4.68 ± 0.76 ^bc^

Notes: SQ, Super Qianhao; NJ5, Nanjing 5; DF, dietary fiber; FOS, fructooligosaccharide; INU, inulin; DDT, dough development time; DST, dough stability time; C1–Cs, protein weakness degree; C3, maximum gelatinization torque; C3–C4, starch breakdown; C3/C4, amylase activity; C5–C4, starch setback; α, heating speed; β, gelatinization speed; γ, enzyme degradation speed. All data are expressed as mean ± SD; number of repetitions: *n* = 3, and each rice variety, each DF species, and each DF addition amount separately had 30, 30, and 12 data points for the GLMU analysis. Different superscript letters indicate significant differences (*p* < 0.05) within the same column for each parameter.

**Table 8:** Effect of adding FOS or inulin on starch crystallinity and protein conformation in cooked japonica rice dough.

Factor	Levels	R_1022/995_	R_1047/1022_	R_1068/1022_	
Rice	SQ	1.033 ± 0.002 ^c^	0.947 ± 0.002 ^abc^	0.857 ± 0.004 ^abc^	
variety	NJ5	1.047 ± 0.002 ^ab^	0.941 ± 0.002 ^de^	0.846 ± 0.004 ^d^	
DF	FOS	1.046 ± 0.002 ^ab^	0.938 ± 0.002 ^ef^	0.842 ± 0.004 ^d^	
species	INU	1.034 ± 0.002 ^c^	0.949 ± 0.002 ^ab^	0.861 ± 0.004 ^ab^	
Addition	0	1.051 ± 0.004 ^a^	0.944 ± 0.003 ^bcd^	0.851 ± 0.004 ^bcd^	
(%)	3	1.029 ± 0.004 ^c^	0.951 ± 0.003 ^a^	0.866 ± 0.004 ^a^	
	5	1.048 ± 0.004 ^a^	0.935 ± 0.003 ^f^	0.829 ± 0.004 ^e^	
	7	1.041 ± 0.004 ^b^	0.942 ± 0.003 ^cde^	0.847 ± 0.004 ^cd^	
	10	1.031 ± 0.004 ^c^	0.947 ± 0.003 ^abc^	0.864 ± 0.004 ^a^	
Factor	Levels	β-Sheet(%)	Random coil(%)	α-Helix (%)	β-Turn (%)
Rice	SQ	54.546 ± 0.070 ^cd^	15.331 ± 0.031 ^b^	15.553 ± 0.035 ^c^	14.570 ± 0.035 ^abc^
variety	NJ5	54.697 ± 0.070 ^b^	15.161 ± 0.031 ^ef^	15.599 ± 0.035 ^bc^	14.543 ± 0.035 ^bc^
DF	FOS	54.522 ± 0.070 ^cd^	15.286 ± 0.031 ^bc^	15.594 ± 0.035 ^bc^	14.598 ± 0.035 ^ab^
species	INU	54.722 ± 0.070 ^b^	15.206 ± 0.031 ^de^	15.558 ± 0.035 ^c^	14.514 ± 0.035 ^cd^
Addition	0	55.043 ± 0.111 ^a^	15.122 ± 0.049 ^f^	15.389 ± 0.055 ^d^	14.446 ± 0.039 ^d^
(%)	3	54.749 ± 0.111 ^b^	15.183 ± 0.049 ^def^	15.539 ± 0.055 ^c^	14.529 ± 0.039 ^bc^
	5	54.233 ± 0.111 ^e^	15.422 ± 0.049 ^a^	15.708 ± 0.055 ^a^	14.638 ± 0.039 ^a^
	7	54.410 ± 0.111 ^de^	15.271 ± 0.049 ^bcd^	15.678 ± 0.055 ^ab^	14.641 ± 0.039 ^a^
	10	54.674 ± 0.111 ^bc^	15.232 ± 0.049 ^cde^	15.566 ± 0.055 ^c^	14.528 ± 0.039 ^bc^

Notes: SQ, Super Qianhao; NJ5, Nanjing 5; DF, dietary fiber; FOS, fructooligosaccharide; INU, inulin; R_1022/995_ and R_1047/1022_ show the degree of short sequences at the surface of starch granules, and R_1068/1022_ shows the interaction between starch and proteins [28]. All data are expressed as mean ± SD; number of trial repetitions: *n* = 3, and each rice variety, each DF species, and each DF addition amount separately had 30, 30, and 12 data points for the GLMU analysis. Different superscript letters indicate significant differences ( *p*< 0.05) within the same column for each parameter.

**Table 9:** The properties of japonica rice samples.

Rice Variety	Moisture Content (%)	Kernel Length -to-Width Ratio	Taste Value (%)	Fatty Acid Value (mgKOH/100 g)	Amylose (%)	Protein(%)
SQ	12.30 ± 0.07 ^b^	1.72 ± 0.01 ^a^	79.31 ± 1.24 ^b^	9.61 ± 0.03 ^a^	15.74 ± 0.48 ^b^	9.51 ± 0.19 ^a^
NJ5	12.01 ± 0.10 ^a^	1.80 ± 0.06 ^a^	86.33 ± 0.58 ^a^	5.48 ± 2.07 ^b^	17.57 ± 0.25 ^a^	8.23 ± 0.15 ^b^

Note: NJ5 is cv. Nanjing 5; SQ is cv. Super Qianhao. The data are given as mean ± SD, number of repetitions: *n* = 3. Different superscript letters indicate the significant differences (*p* < 0.05) in the column.

## Data Availability

The original contributions presented in this study are included in this article, and further inquiries can be directed to the corresponding author.
